# Vitamin D Axis in Inflammatory Bowel Diseases: Role, Current Uses and Future Perspectives

**DOI:** 10.3390/ijms18112360

**Published:** 2017-11-07

**Authors:** Rita Del Pinto, Claudio Ferri, Fabio Cominelli

**Affiliations:** 1 Department of Life, Health and Environmental Sciences, Division of Internal Medicine and Nephrology, University of L’Aquila, 67100 L’Aquila, Italy; ritadelpinto@gmail.com (R.D.P.); claudio.ferri@cc.univaq.it (C.F.); 2 Division of Gastrointestinal and Liver Disease, Case Western Reserve University, Cleveland, OH 44106, USA; 3 Digestive Health Institute, University Hospitals Cleveland Medical Center, Cleveland, OH 44106, USA

**Keywords:** vitamin D receptor, vitamin D metabolism, intracellular signaling, regulation of immune system, analogs of vitamin D, dysbiosis

## Abstract

Increasing evidence supports the concept that the vitamin D axis possesses immunoregulatory functions, with vitamin D receptor (VDR) status representing the major determinant of vitamin D’s pleiotropic effects. Vitamin D promotes the production of anti-microbial peptides, including β-defensins and cathelicidins, the shift towards Th2 immune responses, and regulates autophagy and epithelial barrier integrity. Impairment of vitamin D-mediated pathways are associated with chronic inflammatory conditions, including inflammatory bowel diseases (IBD). Interestingly, inhibition of vitamin D pathways results in dysbiosis of the gut microbiome, which has mechanistically been implicated in the development of IBD. Herein, we explore the role of the vitamin D axis in immune-mediated diseases, with particular emphasis on its interplay with the gut microbiome in the pathogenesis of IBD. The potential clinical implications and therapeutic relevance of this interaction will also be discussed, including optimizing VDR function, both with vitamin D analogues and probiotics, which may represent a complementary approach to current IBD treatments.

## 1. Introduction

Vitamin D is a pleiotropic hormone of the steroid/thyroid superfamily, classically known for calcium homeostasis, but with several, additional non-calcemic effects, ranging from immune modulation to cell differentiation and intercellular adhesion. The active form of the vitamin is obtained after a two-step hydroxylation of the inactive precursor, cholecalciferol. Cholecalciferol undergoes hydroxylation at position 25, and the resultant inactive intermediate calcifediol [25(OH)D3] is further hydroxylated at position 1 to obtain the active vitamin, calcitriol [1,25(OH)2D3]. Both the inactive, as well as the active, forms of vitamin D circulate in the bloodstream bound to vitamin D-binding protein (VDBP). The active form exerts its effects by binding to a specific transcription-regulating molecule, the vitamin D receptor (VDR). Low levels of vitamin D have been associated with a variety of immune-mediated diseases, as well as with altered immune responses to pathogens and increased susceptibility to infection and cancer [1,2,3,4]. In addition, its binding protein has the ability to directly mediate some immunoregulatory functions [5], while its receptor, also expressed on immune cells, participates in modulation of inflammatory pathways [6].

Inflammatory bowel diseases (IBD) are chronic, inflammatory disorders that can affect the entire gastrointestinal tract, and are thought to result from inappropriate and ongoing immune activation in response to gut luminal agents in genetically predisposed individuals [7,8]. Vitamin D deficiency is common in the setting of the two major forms of IBD, Crohn’s disease (CD) and ulcerative colitis (UC) [9,10]. Interestingly, growing evidence supports the concept that intestinal dysbiosis and vitamin D metabolism are related in several ways, which may be of interest for uncovering novel pathogenic mechanisms and for future therapeutic directions in IBD [9,10].

In this context, we reviewed the current literature to summarize emerging evidence regarding the critical role of the vitamin D axis in the setting of IBD, with particular emphasis on the cross-talk between the gut microbiome and Vitamin D/VDR-mediated genetic and immune responses. Our results depict an interesting concept, wherein a balanced intervention on VDR function, both with vitamin D analogues and probiotics, may represent a complementary approach to IBD treatment.

## 2. Why Target the Vitamin D Axis in IBD?

Vitamin D, its binding protein and its receptor, constitute the so-called vitamin D axis, for which many interesting properties at the level of gut physiology have been emerging. The cellular actions of vitamin D are specifically mediated by the VDR, a ligand-dependent transcriptional regulator of the nuclear receptor superfamily, which is expressed in a variety of cell types, including mucosal immune cells and the intestinal epithelium. In addition, the enzyme, Cyp27B1, which converts circulating, inactive vitamin D [25(OH)D3] into its active, VDR-binding form [1,25(OH)2D3], is also expressed in different immune cell populations, as well as the intestinal epithelium. Co-localization of such key players in different cells of the gastrointestinal tract suggests the role of active vitamin D as a paracrine molecule, whose levels are modulated according to local needs [11,12,13]. Interestingly, intestinal bacteria have been shown to regulate the vitamin D axis within the gut, acting on the intestinal epithelium, as well as on local mucosal immune cells. In particular, the expression of Cyp27B1 has been reported to be reduced in intestinal epithelial cells of germ-free and antibiotic-treated mice, as is the expression of several genes involved in innate immunity (e.g., antibacterial peptides, tight junction proteins, cytokines and their receptors), suggesting that the synthesis of active vitamin D by the ‘‘microbiota-dependent’’ Cyp27B1 enzyme may be a requirement for the proper development of local innate immunity [14].

Similarly, probiotics and pathogenic bacteria have shown to modulate VDR expression in opposite directions, with the former increasing [15], and the latter decreasing [16], its expression. In particular, VDR is subject to the actions of antagonist molecules, in an attempt of pathogens to escape immune surveillance and manipulate host genes to increase their own survival [6,17]. The VDR gene (*VDR*, 12q12—14) is among the candidate genes that have been extensively studied for associations with IBD. Results from two recent meta-analyses showed that the risk of CD is increased in the presence of the VDR *ApaI* polymorphism and the *TaqI* tt genotype, whereas the risk of UC may decrease in the presence of the VDR *TaqI* polymorphism, especially in Caucasians [18,19]. For Asians, the VDR *FokI* polymorphism has been associated to susceptibility to UC [19]. In experimental animal models, VDR knockout (VDR KO) mice showed greater susceptibility to experimental colitis, manifested as worse histology scores, increased expression of genes encoding proinflammatory cytokines, and the development of intestinal dysbiosis [9,20,21]. The latter, in turn, was shown to dramatically alter the composition of bile acids in feces, and this may profoundly affect further molecular signaling, with particular focus on the cellular responses involved in immune regulation [22,23].

Vitamin D binding protein (VDBP), or Gc globulin (human group-specific component (Gc)), is a 55 kDa serum protein secreted by the liver and belonging to the albumin superfamily that is responsible for transporting active and inactive vitamin D in the plasma [24]. Single nucleotide polymorphisms (SNPs) in the gene encoding VDBP have been shown to affect circulating levels of this protein, as well as of circulating 25(OH)D3 [25]. VDBP is essential for the proper functioning of the endocytic pathway required for the renal uptake of 25(OH)D3 into renal tubular cells and consequent activation of the vitamin [26]. An association has been reported between specific SNPs in VDBP (VDBP 420 variant Lys; 416 Asp 420 Lys) and IBD, although their exact meaning in the pathogenesis of the disease remains to be determined [27]. VDBP has shown additional properties aside from a vitamin D carrier, particularly serving as a chemotactic and scavenger agent, as well as a macrophage activator. In fact, plasma VDBP effectively scavenges G-actin released at sites of necrotic cells and prevents polymerization of actin in the circulation [24]. In addition, it functions as a co-chemotactic factor for C5a, which is a very potent chemotactic factor for all leukocytes, as well as several other cell types, and is generated by limited proteolytic cleavage of C5 during complement activation [28]. After stepwise modification of its sugar moiety, VDBP is also converted into macrophage-derived macrophage activating factor (GcMAF), which not only produces a fully active ingestion function and cytotoxic capacity in 3 hours [29], but also has additional functions, such as antitumor [30,31,32] and antiangiogenic [33,34,35] activities. As a result, cloned GcMAF constructs [36] and GcMAF-mimicking peptides [37] have been developed for the purpose of studying their potential clinical use as immunopotentiators.

## 3. The Vitamin D Axis, Gut Microbiome, and the Gut Mucosal Immune System: Interplay at the Intestinal Level

Intestinal homeostasis is determined by the interplay among multiple factors, linked through complex molecular signaling, including the intestinal epithelial barrier, the gut microbiome, and components of the innate and adaptive immune systems. Interesting effects of the vitamin D axis on each of these components have been described.

### 3.1. Intestinal Epithelial Barrier

The differentiated intestinal epithelium constitutes a barrier for the free exchange of molecules between the intestinal lumen and the gut mucosa. In fact, the presence of adhesion structures between adjacent epithelial cells, namely tight junctions (occludin, proteins of the zonula occludens, and claudins), adherens junctions (E-cadherin, catenins, nectin [38]), desmosomes and gap junctions, guarantees the sealing of the paracellular space and regulates the permeability of the mucosal barrier. The integrity of the gut mucosa is also crucial for protection against microorganisms. Disruption of barrier function, in fact, facilitates infection with enteropathogenic bacteria and the development of intestinal inflammation [39] and IBD [40,41,42,43,44]. Conversely, probiotics have been shown to decrease paracellular permeability, evaluated by transepithelial electrical resistance (TEER), as well as to decrease epithelial apoptosis, in different models of intestinal inflammation [45,46,47]. Impaired mucosal barrier function with hyperpermeability is also common in the setting of several infectious and immune-mediated diseases of the lung (cystic fibrosis [48], interstitial lung disease [49], asthma [50,51], tuberculosis [48], chronic obstructive pulmonary disease [52]), skin (atopic dermatitis [53]), oral mucosa [54,55] and eyes [56], where impairment of the vitamin D axis has been described. In addition, intestinal epithelial cells cooperate with the hematopoietic compartments for the management of enteric infections and play an essential role in the initiation of type 2 immune responses [57,58]. Examples of epithelial-derived immunocompetent cells, include Paneth cells, goblet cells and the specialized phagocytic, antigen-presenting M cells located in the follicle-associated epithelium overlying organized lymphoid structures.

Vitamin D and its receptor have a protective effect on epithelial barriers in various tissues, including the gut mucosa [59,60,61]. In fact, it is well documented that active vitamin D increases the expression of several tight junction and adherent junction proteins [62]. In particular, active vitamin D induces the expression and/or membrane translocation of occludin, the zonula occludens proteins, ZO-1 and ZO-2, claudins 2, -7 and -12, and vinculin at several anatomic sites, including corneal epithelium, podocytes, and enterocytes [63,64]. In vitro studies demonstrated that pretreatment with 1,25(OH)2D3 protects intestinal epithelial cells from increased permeability induced by dextran sulfate sodium (DSS), and in vivo studies using VDR KO mice showed increased susceptibility to DSS-induced colitis when compared to their wild-type littermates [65]. Moreover, it has been demonstrated that intestinal epithelial VDR signaling plays a key role in maintaining mucosal barrier integrity by suppressing intestinal epithelial cell apoptosis, thus regulating gut mucosal inflammation [59,66]. In addition to their sealing properties, adherent proteins are actively involved in signal transduction, and VDR can regulate such pathways acting on VDR-regulated promoters. For instance, active vitamin D attenuates growth and promotes differentiation in colon cancer cells by the VDR-mediated induction of E-cadherin and inhibition of β-catenin signaling [67,68,69]. Taken together, these data confirm the role of the vitamin D axis in mucosal barrier development, integrity and healing capacity.

### 3.2. Intestinal Microbiome

In recent years, the Human Microbiome Project has provided unprecedented information regarding the diversity and function of microbial communities and their genes, referred to as the human microbiome [70]. Sequencing of microbial ribosomal RNA obtained from different body sites showed that the number and relative distribution of distinct microbial species characterized health and disease states in humans; for instance, decreased diversity in the gut was observed in IBD [71]. The intestinal microbiome has a role in several functions, including metabolism, mucosal barrier physiology, immunity, and inflammatory signaling, and its disruption, or dysbiosis, is associated with the development, maintenance, and perpetuation of various clinical conditions, both intestinal and extraintestinal. By regulating the expression of antimicrobial peptides [72,73], mucosal barrier function, and innate immunity [61], vitamin D and its receptor have been shown to influence the composition and functions of bacterial communities in the gut, protect from dysbiosis, as well as experimental IBD and its symptoms [9,74].

Although there appears to be no unique and optimal composition of the microbiome to promote gut health, *Bacteroides* and *Firmicutes* species are the most highly represented under normal conditions [70]. During IBD, a reduction in the number of species within the phylum *Firmicutes*—specifically the Clostridium clusters XIVa and IV- and *Bacteroidetes*-namely Bifidobacterium, Lactobacillus, and Ruminococcaceae (particularly the butyrate-producing genus Faecalibacterum)—and an increase in *Bacillus* spp and *Enterobacteriacae*, is observed [75,76,77]. Studies using VDR KO mice and wild-type littermates showed defective autophagy and consequent gut dysbiosis in the former, with depletion of fecal *Lactobacillus* and *Bacteroides* [78]. In a colitis model, administration of butyrate, a fermentation product of gut bacteria, increased intestinal VDR expression and suppressed inflammation [78]. Interaction between the vitamin D axis and the gut microbiome was further demonstrated in a model of experimental colitis on CYP27b1 KO and VDR KO mice compared to littermates [9]. Results of this study showed greater susceptibility of KO mice to DSS colitis, which was associated with bacterial imbalance, with more *Proteobacteria* and less *Firmicutes*, similarly to that observed in patients with IBD. Vitamin D deficiency itself was also shown to be a co-factor for dysbiosis in the setting of a high-fat dietary regimen, and this effect was mediated by the downregulation of specific α-defensins from ileal Paneth cells, as well as of tight junction genes in the absence of vitamin D, with consequent endotoxemia and systemic inflammation [79]. Pathogens may also regulate the monocyte/macrophage vitamin D axis in their own favor through DNA methylation on specific sequences, namely micro-RNAs (miRs) [13]. As an example, miR-21 can interact with CYP27B1 mRNA and suppress its activity, thus decreasing localized synthesis of active vitamin D in monocytes [80].

Probiotics, consisting of ingestible non-pathogenic living microorganisms with the ability to confer some beneficial effects to the host when consumed in adequate amounts as food components [81], have been widely used in clinical trials for the treatment of IBD with variable results [82,83]. It has been recently shown that a properly functioning VDR pathway is required for probiotic protection against colitis [84], a finding that is of importance since VDR expression can be significantly decreased in IBD patients as a consequence of chronic inflammation [85] or dysbiosis [6,17]. VDR KO mice, in fact, did not respond to probiotics such as *Lactobacillus rhamnosus* strain GG (LGG) and *Lactobacillus plantarum* (LP) and had worse severity of Salmonella-induced colitis compared to littermates [84]. The same probiotics in wild-type mice, indeed, were able to increase VDR expression and its transcriptional activity, with increased expression of antimicrobial peptides, and had the ability to confer physiological and histologic protection from Salmonella-induced colitis [84]. Taken together, the interplay of the vitamin D axis with the intestinal microbiome is an intriguing, yet undiscovered field of research, with potential clinical implications.

### 3.3. Immune System

Besides its role as a site for nutrient absorption, the gut also hosts a unique immune system, composed of coordinated immunocompetent cells that cooperate in the difficult, unparalleled task of discriminating between harmful and beneficial antigens, among the plethora of diverse antigenic components of its intraluminal content [86]. Such ability is crucial for the induction of tolerance towards nutrients and commensal bacteria, as well as for first-line protection against pathogens. Apart from epithelial-derived immunocompetent cells, the intestinal immune system consists of organized lymphoid structures, such as Peyer’s patches (PPs), cryptopatches and isolated lymphoid follicles (ILFs), which are located immediately below the epithelial layer within the lamina propria. Within the gut mucosa reside several immune populations, including intraepithelial effector lymphocytes interspersed within the epithelial lining, polarized CD4+ T cells, such as T regulatory cells (Treg), T helper 1, 2 and 17 (Th1, Th2, Th17) cells, and IgA-producing plasma cells, as well as innate immune cells with antigen-presenting cell function, such as dendritic cells (DCs) and monocytes/macrophages, and the recently identified heterogeneous group of innate lymphoid cells (ILCs) [87].

The vitamin D axis is an important regulator of the innate and adaptive immune systems. Its effects include decreasing Th1/Th17 T cells and pro-inflammatory cytokines, such as IL-1, IL-6, IL-8, IFNγ, and TNFα, in favor of Th2 response, increasing Tregs, downregulating T cell-driven IgG production, inhibiting DC differentiation, and helping maintain self-tolerance, while enhancing protective innate immune responses [88]. In particular, active vitamin D [89] and glycosylated VDBP [29] boost autophagy in human monocytes/macrophages, with inhibitory effects on intracellular pathogens, including Mycobacterium tuberculosis and human immunodeficiency virus type 1 [90]. By boosting C5a activity, VDBP enhances cell recruitment during inflammation, with particular reference to neutrophils, monocytes and fibroblasts [28,91]. VDBP also modulates the availability of 25(OH)D3 to DCs, indirectly regulating the amount of active vitamin derived from DCs that can become available to T cells [92]. Of importance in IBD, VDR mediates enhanced production of antimicrobial peptides, such as β-defensin 2 (DEFB4/HBD2) and cathelicidin (CAMP) that are traditionally boosted by the activation of nucleotide-binding oligomerization domain-containing protein 2 (NOD2) after stimulation by microbial muramyl dipeptides [73]. Interesting, a vitamin D deficient diet can repress the expression of defensins and their activating enzyme, matrix metalloproteinase 7 (MMP7), with consequent dysbiosis [79]. Notably, the region encoding DEFB2/HBD2 has been identified as a CD susceptibility locus. The NOD2 gene itself has a VDRE [93,94], and mutations or dysregulation in NOD2, with consequent decreased expression of antimicrobial peptides, impaired autophagy, and dysbiosis, are also associated with IBD [93,95,96]. In addition, NOD2 KO-associated altered microbial composition, with greater susceptibility to DSS colitis, was transmissible to co-housed wild-type mice [96]. Another mechanism for vitamin D to activate innate immunity occurs by enhancing the function of another pattern recognition receptor, the Toll-like receptor 4 (TLR4), which is traditionally activated after recognition of lipopolysaccharide (LPS), a cell wall component of gram-negative bacteria [97,98]. Interestingly, VDR levels were found to be reduced by more than 50%, and the pro-inflammatory cytokines, TNFα and IL-1β, were elevated, in colonic biopsies from patients with CD and UC, indicating that VDR can be repressed by inflammatory mediators [59]. Studies on wild-type, specific pathogen-free IL-10 KO mice, and VDR KO mice showed impaired T cell homing to the gut in the absence of vitamin D signaling, with less CD8+ intraepithelial lymphocytes, low levels of IL-10 and consequent increased inflammatory response to the normally harmless commensal flora [99]. Stimulation of VDR by microbial-derived bile acids is another pathway of immune modulation mediated by the vitamin D axis, of which changes in bile acid profiles after dysbiosis can impact such signaling [23].

## 4. Role of the Vitamin D Axis and Dysbiosis in Immune-Mediated Diseases: Focus on Experimental Studies Targeting the Vitamin D Axis in IBD

Autoimmune diseases are self-directed pathologies resulting from an aberrant activation of the immune system against harmless self-antigens, with consequent inflammation and tissue damage compromising the affected target organs or systems. An association has been consistently described between autoimmunity and vitamin D epidemiology and genetics, thus promoting great interest in the potential clinical applications of targeting the vitamin D axis in such conditions, including IBD. Considering the interplay between the vitamin D axis, the gut microbiome and the mucosal immune system, the reciprocal effects of targeted interventions on each of these components represents an intriguing therapeutic opportunity.

Human clinical trials targeting the vitamin D axis in IBD are often heterogeneous in their design and methods, and this prevents the exact comparability of results. However, despite differences in disease history and treatment, as well as additional environmental factors that can influence vitamin D status, most studies are in agreement regarding the beneficial effects of vitamin D supplementation on disease activity and/or quality of life [100,101,102,103,104,105,106]. As an example, a randomized, double-blind placebo-controlled study on 94 CD patients with inactive disease, assigned to either 1200 IU vitamin D3 daily or placebo for 12 months, showed that the relapse rate had a trend towards being lower in the treatment versus placebo group (*p* = 0.06) [100]. Similar results were described after a trial of high-dose vitamin D3 at 10,000 IU daily (*n* = 18) compared to 1000 IU daily (*n* = 16) for 12 months in patients with CD in remission, with less clinical relapse of disease in patients receiving a higher dose of vitamin D (0% vs. 37.5%, *p* = 0.049) [105]. Similarly, a recent prospective randomized controlled trial on 18 patients with UC and hypovitaminosis D showed that vitamin D3 supplementation improved quality of life and reduced UC disease activity, especially at higher doses (4000 IU daily versus 2000 IU daily) [103]. Other studies have reported a short-term beneficial effect on disease activity in CD patients treated with vitamin D, particularly in its active form [101]. A recent pilot study on CD and UC patients assigned to oral vitamin D supplementation targeting a serum concentration of 100–125 nmol/L showed successful and safe improvement of symptom-based activity scores, but did not show significant changes in objective measures of intestinal or systemic inflammation after 12 weeks [104]. In addition, a randomized placebo-controlled clinical trial on 108 IBD patients with vitamin D deficiency (serum 26(OH)D < 30 ng/mL) showed that oral supplementation with cholecalciferol 50,000 UI/week was not significantly efficacious in reducing serum TNFα levels after 12 weeks (*p* = 0.07) [106]. In patients with multiple sclerosis, daily vitamin D supplementation (5000 IU for 3 months) increased the abundance of *Akkermansia*, which promotes immune tolerance, as well as *Faecalibacterium* and *Coprococcus*, which produce the VDR-activating, anti-inflammatory fermentation product butyrate [107]. In a pilot study, the gut microbiome was modified and intraepithelial CD8+ T-cells in the terminal ileum increased, even in healthy volunteers after 2-months of vitamin D supplementation [108].

Given the growing evidence supporting the intimate relationship between the gut microbiome and the vitamin D axis in autoimmunity, potential contributing factors may help to explain the observed results. For instance, bacterial-induced epigenetic modifications in cytochromes involved in vitamin D metabolism, as well as changes in bile acid profiles after dysbiosis, can influence VDR-mediated signaling. Use of probiotics, indeed, promotes VDR expression and its antimicrobial effects, which is beneficial to dampening colonic inflammation. In turn, vitamin D may restore a healthier gut microbiome and attenuate inflammation. Therefore, a correct nutraceutical approach to immune-mediated diseases, including IBD, should contemporarily exert beneficial effects on both VDR expression and signaling, and the gut microbiome.

## 5. Future Directions

The role of vitamin D in immune-mediated diseases appears to be intimately associated with bacteria metabolism, with chronic dysbiosis causing VDR dysfunction and triggering a vicious cycle, wherein a compromised immune system perpetuates disease. Restoring VDR function at different cellular levels should therefore be considered as a therapeutic option. Probiotics and olmesartan proved to be effective in this sense in experimental settings, but require additional testing in human studies in specific clinical settings [15,16,84,109]. To date, in fact, sparse research has been performed on the reciprocal effects of probiotics and vitamin D in humans. An example is a double-blind, placebo-controlled, randomized trial on 127 otherwise healthy hypercholesterolemic adults randomized to consume *L. reuteri* NCIMB 30242 or placebo capsules over a 9 week intervention period. The study showed a significant increase in circulating vitamin D in response to oral probiotic supplementation compared to placebo (*p* = 0.003) [110]. Olmesartan is an angiotensin receptor blocker with VDR-binding properties. According to some evidence, it acts as a VDR agonist and is able to restore proper VDR function by displacing bacterial products bound to the receptor with inhibitory effects [16]. Olmesartan was proposed in combination with pulsed, low dose, broad-spectrum, bacteriostatic antibiotics as an approach to reverse the disease process in autoimmune diseases [16]. Raising epithelial VDR levels by vitamin D analogues or by anti-TNF therapy may represent an additional mechanism to ameliorate IBD by reducing IEC apoptosis [59]. In addition, several VDR ligands with low calcemic effects, but high therapeutic potential have drawn attention as possible alternatives to active vitamin D [111]. In fact, despite major side effects of vitamin D supplementation, like hypercalcemia, have been rarely reported and are usually only observed after exposure to high doses of the active hormone, the risk of vascular calcifications, hypercalciuria and renal complications following long-term exposure to vitamin D remains uncertain [112]. Non-vitamin D VDR ligands demonstrated in vivo efficacy in protecting against, or reducing the severity of, experimental colitis [113,114,115,116]. Similarly, in vitro studies on human immune cells have proven the ability of these analogues to modulate the immune system through the switch of Th1 into Th2 immune response and the down-regulation of pro-inflammatory cytokines in peripheral mononuclear cells [117,118,119,120]. Glucuronide conjugates of vitamin D represent an additional option for targeted delivery of active vitamin D at specific sites of the gastrointestinal tract, namely the ileum and colon, a mechanism that exploits bacteria metabolism. In fact, bacteria residing in the lower gastrointestinal tract produce β-glucuronidase enzymes that can cleave glucuronide and liberate 1,25(OH)2D3 for local actions [121,122]. Duodenal bacteria did not appear to produce the same enzyme [122]. Therefore, the oral administration of such water-soluble vitamin D compounds allows their selective action at the lower intestinal tract, which is typically affected by IBD, without hypercalcemic effects, and theoretically improves the efficacy of rectally-administered vitamin D, whose diffusion would exclude the ileum. In accordance with this, βGluc-1,25(OH)2D3 proved to ameliorate the severity of experimental IBD in mice, without rising blood concentrations of calcium [122]. Finally, in the era of personalized medicine, a better knowledge of targeting gene expression, or anticipating the potential response to treatment based on genetic variants of specific genes [123], may further help improving quality of life in chronic diseases.

In conclusion, the horizon opened by current advances in knowledge in the field of microbiomics and nutraceuticals depicts interesting implications in the treatment of immune-mediated diseases. Despite persistent gaps preventing the possibility of recommendations to incorporate manipulation of the vitamin D axis and microbiome into clinical practice guidelines, results of recent research encourage the pursuit of this goal for better, targeted therapy for the treatment of patients with IBD.

## Abbreviations

VDBP	Vitamin D binding protein
VDR	Vitamin D receptor
IBD	Inflammatory bowel diseases
CD	Crohn’s disease
UC	Ulcerative colitis
KO	Knockout
SNPs	Single nucleotide polymorphisms
GcMAF	Group-specific component macrophage activating factor
TEER	Transepithelial electrical resistance
IECs	Intestinal epithelial cells
DSS	Dextran sodium sulfate
RNA	Ribonucleic acid
DNA	Deoxyribonucleic acid
PPs	Peyer’s patches
ILFs	Isolated lymphoid follicles
Treg	Regulatory T cells
Th	T helper
DCs	Dendritic cells
ILCs	Isolated lymphatic cells
IL	Interleukin
IFN	Interferon
TNF	Tumor necrosis factor
HBD	Human beta defensin
CAMP	Cathelicidin antimicrobial peptide
NOD2	Nucleotide-binding oligomerization domain-containing protein 2
MMP	Matrix metalloproteinase
TLR	Toll like receptor
LPS	Lipopolysaccharide
IU	International unit
OR	Odds ratio
CI	Confidence interval
